# Vitamin D_3_ Status and the Association with Human Cathelicidin Expression in Patients with Different Clinical Forms of Active Tuberculosis

**DOI:** 10.3390/nu10060721

**Published:** 2018-06-04

**Authors:** Senait Ashenafi, Jolanta Mazurek, Anders Rehn, Beede Lemma, Getachew Aderaye, Amsalu Bekele, Getachew Assefa, Menberework Chanyalew, Abraham Aseffa, Jan Andersson, Peter Bergman, Susanna Brighenti

**Affiliations:** 1 Center for Infectious Medicine (CIM), F59, Department of Medicine, Karolinska Institutet, Karolinska University Hospital Huddinge, 141 86 Stockholm, Sweden; jolanta.mazurek@gmx.com (J.M.); anders.rehn@bredband2.com (A.R.); jan.andersson@ki.se (J.A.); susanna.brighenti@ki.se (S.B.); 2 Department of Surgery, Faculty of Medicine, Black Lion University Hospital and Addis Ababa University, P.O Box: 28287/1000, Addis Ababa, Ethiopia; bedelemma@yahoo.com; 3 Department of Internal Medicine, Faculty of Medicine, Black Lion University Hospital and Addis Ababa University, P.O Box: 28287/1000, Addis Ababa, Ethiopia; getadera@yahoo.com (G.A.); amsalubekele2016@gmail.com (A.B.); 4 Department of Radiology, Faculty of Medicine, Black Lion University Hospital and Addis Ababa University, P.O Box: 28287/1000, Addis Ababa, Ethiopia; g_asefa@yahoo.com; 5 Armauer Hansen Research Institute (AHRI), P.O. Box 1005, Addis Ababa, Ethiopia; menberework@gmail.com (M.C.); aseffaa@gmail.com (A.A.); 6 Department of Medicine, Division of Infectious Diseases, Karolinska University Hospital Huddinge, 141 86 Stockholm, Sweden; 7 Department of Laboratory Medicine (Labmed), Division of Clinical Microbiology, Karolinska Institutet, 141 86 Stockholm, Sweden; peter.bergman@ki.se

**Keywords:** tuberculosis, clinical, vitamin D, antimicrobial, LL-37

## Abstract

Low vitamin D (vitD_3_) is one of the most common nutritional deficiencies in the world known to be associated with numerous medical conditions including infections such as tuberculosis (TB). In this study, vitD_3_ status and its association with the antimicrobial peptide, human cathelicidin (LL-37), was investigated in Ethiopian patients with different clinical forms of TB. Patients with active TB (*n* = 77) and non-TB controls (*n* = 78) were enrolled in Ethiopia, while another group of non-TB controls (*n* = 62) was from Sweden. Active TB included pulmonary TB (*n =* 32), pleural TB (*n* = 20), and lymph node TB (*n* = 25). Concentrations of 25-hydroxyvitamin D_3_ (25(OH)D_3_) were assessed in plasma, while LL-37 mRNA was measured in peripheral blood and in samples obtained from the site of infection. Median 25(OH)D_3_ plasma levels in active TB patients were similar to Ethiopian non-TB controls (38.5 versus 35.0 nmol/L) and vitD_3_ deficiency (<50 nmol/L) was common in both groups (73%). Ethiopians (low latitude) had significantly lower 25(OH)D_3_ levels compared with Swedish non-TB controls (51.0 nmol/L, high latitude), but vitD_3_ status was not affected by tuberculin-positivity or HIV infection. Patients with local lymph node TB had significantly higher 25(OH)D_3_ levels compared with pulmonary TB patients (48.0 versus 29.0 nmol/L). Moreover, plasma 25(OH)D_3_ levels correlated with local LL-37 expression in granulomatous lesions in TB infected lymph nodes. Instead, systemic LL-37 mRNA expression in blood cells was elevated compared with the site of infection in pulmonary and pleural TB. Low vitD_3_ status may be associated with an enhanced peripheral expression of LL-37 in patients with intrathoracic TB that could result from chronic inflammation.

## 1. Introduction

Although tuberculosis (TB) is an ancient disease, it continues to be an enduring danger to society and remains the main cause of death from infectious diseases worldwide [1]. The susceptibility to *Mycobacterium tuberculosis* (Mtb), the causative agent of TB, as well as the course of TB disease is affected by both host- and bacteria-specific factors. Vitamin D_3_ (vitD_3_) status has been suggested to be associated with TB susceptibility and disease progression. The active form of vitD_3_ is a steroid hormone with immunomodulatory properties and vitD_3_ deficiency (VDD) has been associated with impaired control of Mtb infection [2,3]. VitD_3_ is produced in the skin as a result of a direct exposure to sunlight, but can also be obtained via food intake or supplements [4]. Due to the importance of UVB light for the production of vitD_3_, VDD is subjected to seasonal variations [5,6]. It has been observed that VDD, defined as the concentration of 25-hydroxyvitamin D_3_ (25(OH)D_3_) below 50 nmol/L [7], was more common in dark-skinned people as strong skin pigmentation absorbs UVB sunlight and decreases the production of vitD_3_ in the skin [8]. Accordingly, it has been reported that ethnicity was associated with a higher susceptibility to active TB [3,9].

VitD_3_ has the ability to regulate multiple axes of the innate, as well as the adaptive, host immune response [10]. Induction of host defense peptides, such as human cathelicidin (LL-37), in phagocytes and epithelial cells is one of the specific functions ascribed to vitD_3_ [11]. LL-37 has both antimicrobial and immunoregulatory activities, such as direct killing of bacteria via osmotic lysis, but also induction of autophagy, regulation of chemokine production and chemokine receptor expression, modulation of cytokine secretion and chemotactic effects on immune cells [12]. Several immune cell types, including neutrophils and alveolar macrophages, respond to Mtb infection by producing LL-37 in vitro [13,14], although it has also been shown that Mtb could suppress baseline LL-37 expression in human monocyte-derived macrophages [15]. Early during Mtb infection, LL-37 contributes to the control of bacterial growth and several studies have demonstrated that vitD_3_ enhanced the expression of LL-37 and intracellular Mtb killing in macrophages in vitro [11,14,15]. Upon progression of TB disease, vitD_3_, as well as LL-37 itself, may contribute to dampening an excessive inflammatory response to Mtb by reducing the production of pro-inflammatory cytokines and promoting anti-inflammatory responses [16,17,18,19].

Despite a well-established role of vitD_3_ in TB pathogenesis, it is still unknown how 25(OH)D_3_ levels and LL-37 expression correlate with various forms of clinical TB. To address this question, we assessed vitD_3_ status in TB patients and non-TB controls from an HIV-prevalent setting in Addis Ababa, Ethiopia, which is ranked seventh among high TB-burden countries. Plasma levels of 25(OH)D_3_ in Ethiopian patients with different clinical forms of TB including pulmonary TB (PTB), pleural TB and lymph node TB (LNTB) were compared with non-TB controls from Ethiopia, as well as a non-TB control cohort from a low-endemic setting in Stockholm, Sweden. The expression of LL-37 mRNA was assessed in peripheral blood and in cells obtained from the sites of Mtb infection and related to patients’ vitD_3_ status.

## 2. Materials and Methods

### 2.1. Study Participants

TB patients and non-TB controls were recruited in December 2007 to December 2008 at the Chest Unit, Department of Internal Medicine, Black Lion University Hospital, Addis Ababa, Ethiopia, after providing signed informed consent [20,21]. Inclusion criteria: over 18-year old, HIV-negative or HIV-positive, sputum smear-negative patients with clinical symptoms and a clinical diagnosis of active TB. Exclusion criteria: history of previous TB, miliary TB, antimicrobial treatment longer than a week, antiretroviral treatment and a refusal to undergo HIV screening. Enrolled patients with active TB (*n* = 77) had either PTB (*n* = 32), pleural TB (*n* = 20) or LNTB (*n* = 25). PTB was confirmed by a positive Mtb culture of bronchoalveolar lavage (BAL), while pleural TB was confirmed by typical cyto- and histopathology of pleural fluid and biopsy samples and LNTB was similarly confirmed by typical histopathology of lymph node biopsy samples. Clinical TB diagnosis was based on: (1) assessment of the typical clinical TB symptoms lasting over four weeks, including persistent cough, general illness symptoms, such as fever, sweating, and weight loss; (2) pulmonary infiltrations/lesions or pleural effusions revealed by the chest X-ray examination; (3) occurrence of chronic non-tender cervical lymphadenopathy lasting over six weeks; (4) positive response to anti-TB chemotherapy. The Ethiopian non-TB control group (*n* = 78) consisted of both HIV-negative and HIV-positive individuals without any clinical symptoms of active TB. Additional non-TB controls used for mRNA expression analyses at the site of Mtb infection in the lung, pleura, and lymph nodes included patients with a minor hemoptysis (*n* = 10), patients with other non-TB pleural diseases (*n* = 5) and patients with non-specific lymphadenitis (*n* = 5). The Swedish non-TB control group (*n* = 62), consisted of HIV-negative individuals who were recruited at the Immunodeficiency Unit, Karolinska University Hospital, Huddinge, Sweden, as part of a previously described study [22]. Inclusion criteria: 18–75-year old and an increased susceptibility to respiratory tract infections defined as >42 days with symptoms from the respiratory tract during a 12-month period prior to study inclusion. Exclusion criteria: prophylactic antibiotic therapy, history of hypercalcemia or stones in the urinary tract, sarcoidosis, ongoing vitD_3_ supplementation, HIV infection, and pregnancy.

The study was approved by ethical review boards in Ethiopia including the National Research Ethics Review Committee (NRERC), the Institutional Review Board (IRB) and the AHRI/ALERT Ethics Committee and also by the local ethical review board in Stockholm, Sweden (EPN) (registration numbers 2007/675-31/4 and 2009/1678-31/4). 

### 2.2. Patient Materials

Plasma and peripheral blood mononuclear cells (PBMCs) were isolated from blood samples obtained from the Ethiopian study subjects at the time of diagnosis to assess 25(OH)D_3_ levels in plasma and LL-37 mRNA expression in PBMCs. Part of the blood samples were used for HIV testing. Plasma was aliquoted and stored at −80 °C for vitD_3_ analysis. Five million PBMCs were resuspended in 300 μL of RNAlater (Life Technologies, Invitrogen, Carlsbad, CA, USA) and stored at −80 °C for mRNA analysis. BAL fluid was collected from PTB patients with abnormal chest X-ray results using bronchoscopy as previously described [20]. Similarly, pleural fluid was collected from the pleura cavity of patients with pleural TB using thoracentesis [20]. BAL and pleural fluid aliquots were used for diagnostic purposes (Mtb-culture from BAL, and cytology from pleural fluid), while the remaining fluids were centrifuged at 1400 rpm for 25 min to pellet and resuspend the cells in RNAlater. Cell samples were stored at −80 °C for subsequent mRNA analysis. Lymph node biopsies were obtained through surgical removal of one lymph node in the cervical area from patients with a suspected LNTB. One part of the biopsy was used for diagnostic purposes (histopathology) and the remainder was snap frozen and stored at −80 °C for mRNA and immunohistochemical analysis.

### 2.3. Tuberculin Skin Test (TST)

The tuberculin skin test was applied to assess delayed type hypersensitivity towards Purified Protein Derivative (PPD). A total of 0.1 mL of PPD (5TU, SSI, Copenhagen, Denmark) was injected intradermally in the volar aspect of the forearm. The transverse diameter of induration in the injection site was measured by trained nurses 48–72 h later. A positive TST result was recorded when the diameter exceeded 10 mm in HIV-negative individuals, and 5 mm in HIV-positive individuals.

### 2.4. QuantiFERON-TB Gold In-Tube

IFN-γ-release assay that measures IFN-γ production by T cells in vitro, after stimulation of whole blood samples with the Mtb-specific antigens, CFP-10, ESAT-6, TB7.7, according to the manufacturer’s (Cellestis; SSI, Valencia, CA, USA) instructions.

### 2.5. 25(OH)D_3_ Concentrations in Plasma

Plasma samples were collected from the study subjects and vitD_3_ status was determined by assessment of 25(OH)D_3_ concentrations in plasma using the DiaSorin assay conducted at the Chemical Laboratory, Karolinska University Hospital, Solna, Sweden. To demonstrate seasonality of vitD_3_ status, the 25(OH)D_3_ concentrations in TB patients and Swedish non-TB controls, were subdivided into two seasons: June–November and December–May. Ethiopian TB patients were sampled at the time of diagnosis, while longitudinal samples were obtained from the Swedish non-TB controls. Thus, matched samples were obtained from the Swedish non-TB controls in June–November (average 25(OH)D_3_ concentration from two samples per individual) and December–May (average 25(OH)D_3_ concentration from three samples per individual).

### 2.6. mRNA Extraction and Quantitative Real-Time PCR

RNA was extracted from cell samples (5 × 10^6^) or frozen lymph node tissue sections (2 × 50 µm) using the Ambion RiboPure extraction kit (Invitrogen) according to the manufacturer’s instructions. RNA was reverse transcribed to cDNA using superscript reverse transcriptase (SuperScript VILO cDNA master mix, Invitrogen). Amplification of target genes from cDNA was performed using ABI PRISM 7700 sequence detection system, and primer and probe sequences targeting the LL-37 gene were commercially purchased from Applied Biosystems (HS 00189030_m1) (Foster City, CA, USA). 18S was tested together with a panel of commonly used house-keeping genes and was selected as the calibrator as its expression was constitutive and stable in both test and control samples. Hence, Ct (cycle threshold) values were normalized to 18S to provide the delta Ct values. The relative mRNA expression was determined using the Livak method [23]. The data was analysed using 7500 software v2.0.6 (Applied Biosystems, Foster City, CA, USA) and presented as fold change of mRNA in the TB-positive groups (TB and TB/HIV co-infected) in relation to average Ct value recorded for non-TB controls.

### 2.7. Immunohistochemistry and Quantitative Image Analysis of Frozen Tissue Sections

Cryopreserved lymph node tissue biopsies were embedded in OCT-compound (Tissue-TEK, Sakura, Torrance, CA, USA) and cut into 8 µm-thick sections, mounted on HTC microscope slides (Histolabs, Gothenburg, Sweden) and fixed in 4% formaldehyde (Sigma, Stockholm, Sweden) for 15 min. The primary antibody used was polyclonal rabbit anti-human LL-37 (PA-LL37100; Innovagen, Lund, Sweden) and the biotinylated secondary antibody was swine anti-rabbit F(ab^’^)_2_ (Dako, Glostrup, Denmark). Immunohistochemistry (IHC) was performed according to the ABC-method as previously described [24]. Positive staining was developed using a diaminobenzidine substrate (Vector Laboratories, Burlingame, CA, USA) while haematoxylin was used for nuclear counterstaining. Acquired computerized image analysis was applied to quantify IHC stainings in situ using light microscopy (Leica Microsystems, Germany) as previously described [25]. Positive immunostaining was quantified at the single-cell level in 25 to 50 high-power fields using a Qwin 550 software program (Leica Imaging Systems, Wetzlar, Germany). Protein expression was determined as the percent positive area of the total relevant cell area. Tissue sections stained with secondary antibodies only were used as negative controls.

### 2.8. Statistical Analysis

The data were analyzed using a non-parametric Kruskal-Wallis test with Dunn’s post-test (comparing more than two unmatched groups) or the Mann-Whitney test (comparing two unmatched groups). A *p*-value < 0.05 was considered statistically significant. Correlation analyses were conducted using the Spearman’s correlation test. Statistical analysis and preparation of graphs were performed using the GraphPad Prism software, v.7.03 (GraphPad Software, La Jolla, CA, USA).

## 3. Results

### 3.1. VitD_3_ Levels in Plasma from Ethiopian TB Patients as Well as Non-TB Controls were Significantly Lower Compared to Swedish Non-TB Controls

The demographics of the study participants are shown in Table 1. We performed a post hoc analysis of previous studies including Ethiopian patients with active TB (*n* = 77) and non-TB controls from Ethiopia (*n* = 78) [20,21] or non-TB controls from Sweden (*n* = 62) [22]. Gender, age, and numbers of HIV-infected subjects were similar between the Ethiopian groups. In the Swedish non-TB control group, we observed a relatively higher proportion of females and a higher median age compared with the Ethiopian cohort. Fewer TST-positive cases were also found among Ethiopian non-TB controls compared with active TB, which was expected. Interestingly, weight loss was relatively lower in the LNTB group compared with PTB and pleural TB. Furthermore, the occupational level was generally low among active TB patients, between 30% and 53%.

**Figure 1:**
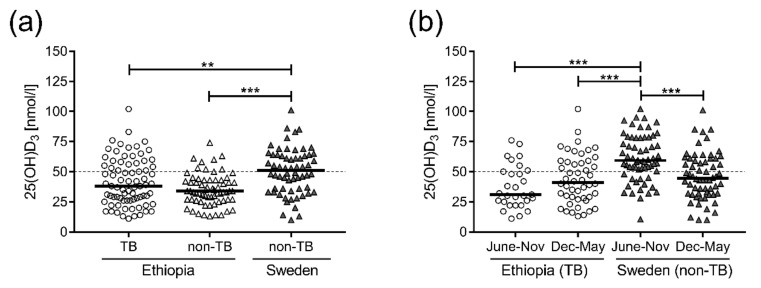
VitD_3_ levels in patients with active TB compared to non-TB control groups. (**a**) Concentrations of 25(OH)D_3_ in plasma from Ethiopian TB patients (circles) and non-TB controls (open triangles) as well as Swedish non-TB controls (closed triangles). VitD3 concentrations for each Swedish control were calculated from an average of five measured time-points. (**b**) Seasonal variations in 25(OH)D_3_ plasma levels in Ethiopian TB patients (circles) and Swedish non-TB controls (closed triangles). Ethiopian TB patients were grouped depending on the time-point of sampling, June–November or December–May. The Swedish controls were sampled at five time-points throughout the year, and thus each individual data represents the average 25(OH)D_3_ concentration assessed at these different occasions, June-November (three measurements) or December–May (two measurements). Data are presented in box plots with horizontal bars indicating the median. ** *p* < 0.01, *** *p* < 0.001. The dotted line is the threshold for VDD, 25(OH)D_3_ < 50 nmol/L.

The study subjects were tested for 25(OH)D_3_ levels in plasma, showing that most of the Ethiopian TB patients as well as the non-TB controls were VDD (73%), defined as 25(OH)D_3_ below 50 nmol/L (Table 1 and Figure 1a). Interestingly, we found no difference in vitD_3_ levels between TB patients and Ethiopian non-TB controls, while 25(OH)D_3_ levels in the Swedish controls were significantly higher compared to both active TB (*p* = 0.008) and Ethiopian controls (*p* < 0.0001) (Figure 1a). VDD was also common in the Swedish cohort (50%), although most deficient cases were sampled during winter time (December–May) (Figure 1b). Accordingly, 25(OH)D_3_ levels assessed from June to November were significantly higher (*p* = 0.001) in the Swedish controls (Figure 1b). A similar, but not significant, trend of relatively lower 25(OH)D_3_ levels around the rainy season (June–November) was also observed in the Ethiopian TB patients (Figure 1b).

### 3.2. VitD_3_ Status in Ethiopian TB Patients and Non-TB Controls was not Affected by Latent TB or HIV Infection but was Associated with the Clinical Form of TB Disease

**Figure 2:**
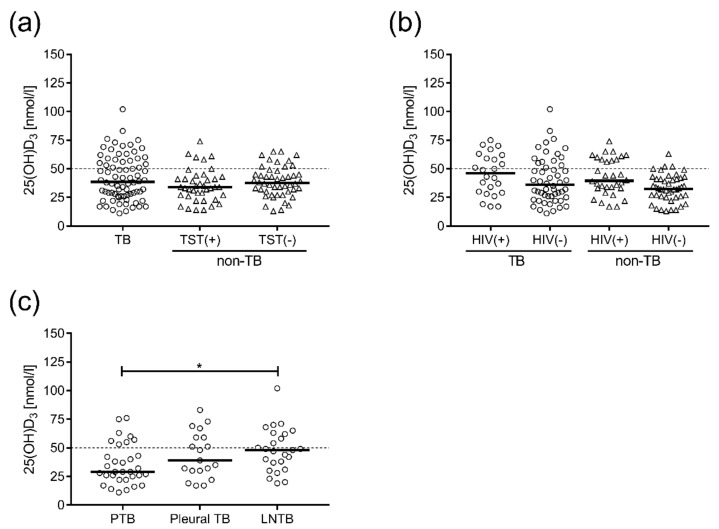
VitD_3_ levels in subgroups of Ethiopian TB patients and non-TB controls. Concentrations of 25(OH)D_3_ in plasma from (**a**) TB patients (circles) compared to non-TB controls (triangles) grouped into TST-positive (+) and TST-negative (−) individuals, (**b**) HIV-positive (+) and HIV-negative (−) TB patients (circles) and non-TB controls (triangles), (**c**) patients with pulmonary TB (PTB), pleural TB, or lymph node TB (LNTB). Data are presented in box plots with horizontal bars indicating the median. * *p* < 0.05. The dotted line is the threshold for VDD, 25(OH)D_3_ < 50 nmol/L.

Next, we compared vitD_3_ status in active TB patients to non-TB controls depending on their TST or HIV status (Figure 2a). All TST-positive individuals had a positive Quantiferon-TB Gold test result, measuring Mtb-specific production of IFN-γ, which suggested that these subjects had latent TB (data not shown). Overall, no differences in vitD_3_ levels were observed comparing these groups (Figure 2a). Neither could we detect any significant differences in vitD_3_ comparing active TB with non-TB controls grouped into HIV-negative and HIV-positive individuals (Figure 2b). However, there was a tendency towards higher vitD_3_ levels in TB/HIV co-infected patients as well as in HIV-positive patients without TB as compared to HIV-negative patients (Figure 2b). Finally, we also grouped the TB patients into the different clinical forms of TB disease: PTB (*n* = 32), pleural (*n* = 20) and LNTB (*n* = 25). Interestingly, patients with PTB had significantly lower (*p* = 0.034) vitD_3_ levels than patients with extra-pulmonary LNTB, while pleural TB patients had intermediate vitD_3_ levels (Figure 2c).

### 3.3. LL-37 mRNA Expression was Elevated in the Peripheral Blood of TB Patients Compared to non-TB Controls and also Systemically in PTB and Pleural TB Compared to the Site of Mtb Infection

**Figure 3:**
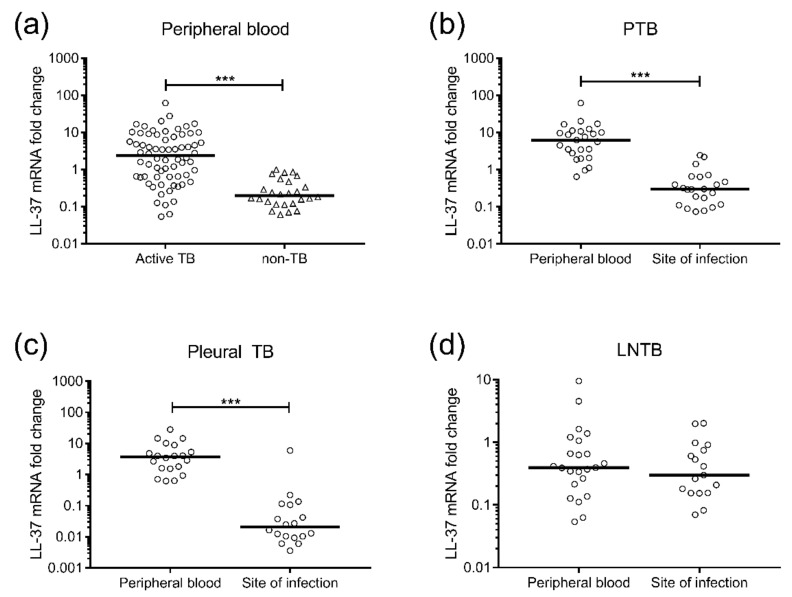
Peripheral and local mRNA expression of the antimicrobial peptide LL-37 in patients with active TB. Relative mRNA expression (median) of LL-37 in (**a**) peripheral blood (PBMCs) from active TB (PTB, pleural TB and LNTB) patients (circles) and non-TB controls (triangles), (**b**) in peripheral blood (PBMCs) compared to the site of infection (BAL cells) from patients with pulmonary TB (PTB), (**c**) in peripheral blood (PBMCs) compared to the site of infection (pleural fluid cells) from patients with pleural TB, and (**d**) in peripheral blood (PBMCs) compared to the site of infection (cells from lymph node tissue) from patients with lymph node TB (LNTB). Data are presented in box plots as a fold change LL-37 mRNA in TB patients normalized to non-TB controls. *** *p* < 0.001.

LL-37 mRNA expression was assessed in PBMCs from TB patients and non-TB controls and also in cells obtained from the site of Mtb infection. There was a significant increase (*p* < 0.001) in LL-37 mRNA in PBMCs from active TB compared with non-TB controls (Figure 3a). Moreover, comparing LL-37 mRNA expression in peripheral blood to the site of infection, revealed significantly reduced (*p* < 0.001) levels at the site of Mtb infection in both PTB (BAL cells) and pleural TB (pleural fluid cells) (Figure 3b,c), while there was no difference in LL-37 mRNA comparing PBMCs to lymph node tissue from patients with LNTB (Figure 3d). 

### 3.4. LL-37 Expression in Mtb-Infected Lymph Nodes Correlated to Plasma levels of 25(OH)D_3_

**Figure 4:**
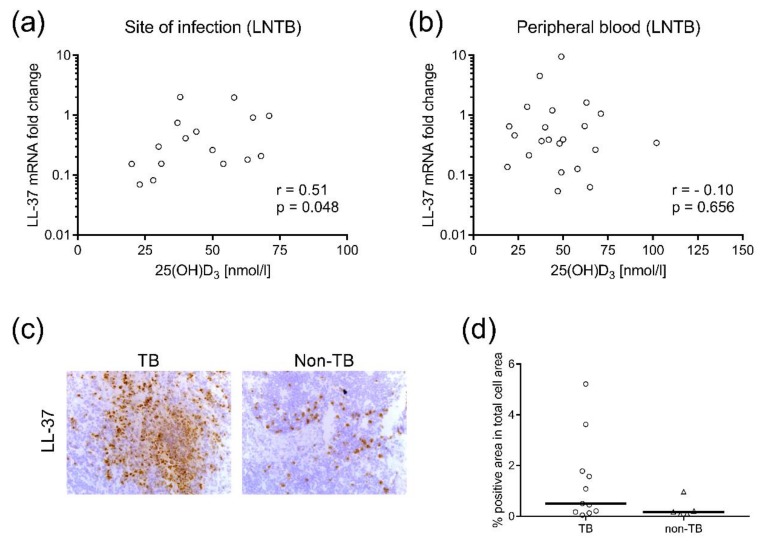
Local expression of LL-37 in patients with lymph node TB (LNTB). Correlation analyses were performed in order to assess the association between plasma concentrations of 25(OH)D_3_ and LL37 mRNA expression (**a**) at the local site of infection (lymph node tissue) and (**b**) in the peripheral blood (PBMCs) of patients with LNTB. (**c**) Representative immunohistochemical images show the expression of LL-37 in granulomatous lymph node tissue from a LNTB patient compared to a non-TB control (magnification × 125). Positive cells are depicted in brown, whereas negative cells were counterstained with hematoxylin in blue. (**d**) In situ computerized image analysis was used to determine LL-37 protein expression (median) in LNTB patients (*n* = 11) compared to non-TB controls (*n* = 5).

We observed a positive correlation between LL-37 mRNA levels assessed in Mtb-infected lymph node samples and 25(OH)D_3_ levels in plasma obtained from LNTB patients (Figure 4a). There was no correlation in the corresponding PBMC samples from these patients (Figure 4b). No LL-37 mRNA/25(OH)D_3_ correlations were detected in PTB or pleural TB (data not shown). These results may imply that plasma levels of vitD_3_ in patients with LNTB (Figure 2c) are associated with LL-37 levels at the local site of Mtb infection, but not in peripheral blood. Accordingly, we quantified protein expression of LL-37 in lymph node tissues from LNTB patients and non-TB controls using microscopy and computerized image analysis (Figure 4c,d). Local expression of LL-37 in LNTB was focused to granulomatous areas rich in neutrophils (cells with segmented nuclei [26]) while the expression in non-TB controls was lower and more randomly scattered (Figure 4c). Image analysis confirmed that protein expression of LL-37 was relatively higher in LNTB compared to the controls (Figure 4d), although there were large individual variations in LL-37 expression in the tissue. Altogether, these results may suggest that LL-37 expression is predominantly localized in the granulomas and, to a lesser extent, in the surrounding tissue.

## 4. Discussion

VDD is common in dark-skinned individuals living in Africa and is associated with many disease conditions, including an elevated risk to develop active TB [4]. In the present study, we demonstrate that Ethiopian patients with active TB were mostly VDD, and that VDD was not more common in TB patients compared with non-TB controls. Overall, 25(OH)D_3_ levels were significantly higher in non-TB controls from Sweden, but only in plasma samples collected during summer time (June-November). Accordingly, there was a pronounced seasonal variability in vitD_3_ status in this group. VitD_3_ status in the Ethiopian cohort was not affected by either TST-positivity or HIV status. Patients with LNTB had higher 25(OH)D_3_ levels in plasma compared to PTB and pleural TB, which correlated with increased expression of LL-37 mRNA at the site of Mtb infection in the lymph nodes. In contrast, patients with PTB and pleural TB showed elevated mRNA levels of LL-37 in peripheral blood compared to the site of infection, which may suggest that LL-37 in the circulation is rather a biomarker of active disease than representing an effective antimicrobial response at the local site. 

A meta-analysis of observational studies from 1980–2006, concluded that there was a 70% probability that a healthy individual would have higher 25(OH)D_3_ serum level than a patient with untreated TB [27]. A later meta-analysis found that the risk of active TB is dependent on the degree of VDD, as vitD_3_ levels ≤25 nmol/L was significantly associated with TB, the range 26–50 nmol/L was associated with an intermediate risk for TB and the range 51–75 nmol/L showed no such association [28]. A systematic review mapping the extent of VDD in Africa, confirmed that VDD and vitD_3_ insufficiency is prevalent in African TB patients [29]. However, a recently performed meta-analysis failed to show any association between VDD and an increased risk of TB in either HIV-negative or HIV-positive Africans [30]. Apparently, vitD_3_ status can vary considerably between different latitudes, populations, ethnic groups, melanin pigmentation and season of sampling. Accordingly, case-control studies from the African continent have shown that TB patients from Ethiopia [31,32] and South Africa [31] were mostly VDD (<50 nmol/L) and also had lower vitD_3_ levels than non-TB controls. Similarly, VDD in individuals with latent TB enhanced the risk of TST conversion [30], whereas sufficient 25(OH)D_3_ levels (>75 nmol/L) might protect against TST conversion [33]. In addition, sputum-conversion after two months of standard anti-TB treatment was less likely in children with intrathoracic TB (PTB and/or pleural TB) who were VDD or vitD_3_ insufficient compared to vitD_3_ sufficient children [34]. In contrast, studies from Zimbabwe [35], Gambia [36] and Tanzania [37] demonstrated that PTB patients with a relatively low proportion of VDD, had higher vitD_3_ levels than non-TB controls. Another study from Guinea-Bissau [38] found that VDD was not more frequent in PTB patients than in healthy controls, but the mean 25(OH)D_3_ concentration remained lower. Importantly, mean 25(OH)D_3_ concentrations varied significantly in these studies from 30–83 nmol/L and severe VDD (<25 nmol/L) was rare in most of these populations [35,36,38]. Combined, these studies support the assumption that active TB is associated with vitD_3_ status and that individuals with VDD are at higher risk to develop the disease [28].

VitD_3_ status is much dependent on seasonal variations, which is characteristic for high latitudes where both the quantity and quality of sunlight (UVB) differs considerably between winter and summer time and also compared to countries at low latitudes [39]. Accordingly, several studies have reported a seasonal pattern of TB incidence that correlated to low vitD_3_ levels [39,40,41,42]. Although our study showed no difference in vitD_3_ status between Ethiopian TB patients and non-TB controls, there was a trend of seasonality in this cohort with lower vitD_3_ levels around the rainy season (June-November). As expected, the seasonality of vitD_3_ status was more pronounced in the Swedish non-TB control group with significantly improved vitD_3_ status around summer time (June-November). Overall, vitD_3_ status in individuals from Stockholm, Sweden, located at the high latitude (59° N) was significantly higher compared to both active TB patients and non-TB controls in Addis Ababa, Ethiopia, at low latitude (9° N). This is consistent with results from a small cross-sectional study, revealing that 25(OH)D_3_ concentrations were significantly higher in both non-pregnant and pregnant Norwegian females compared to Ethiopian females in samples obtained just after the corresponding winter season [43]. Thus, despite sunshine throughout the year in Ethiopia, several studies have shown that VDD is very common [31,32,43]. While variations in sun exposure and skin pigmentation cannot explain differences in low vitD_3_ status in Ethiopians compared to other African populations, other factors could be of importance including diet (no food supplements) or behavior, such as avoiding direct sunlight and primarily indoor activities, especially during the rainy season [29].

Similarly to other reports, we found that vitD_3_ levels in Ethiopians were not influenced by HIV status [6,35,41,44]. It has previously been described that a high proportion of TB/HIV co-infected South African patients were VDD and severe VDD was more common compared with active TB in HIV-negative patients, although VDD was associated with an enhanced TB susceptibility in both HIV-negative and HIV-positive individuals [31]. In line with our results, a small case-control study in Botswana showed that 78% of HIV-infected cases were VDD, however, no difference in vitD_3_ status was detected comparing HIV patients with or without active PTB, suggesting that low vitD_3_ levels are not a major risk factor of HIV-infected as compared with HIV-uninfected individuals [45]. Contrarily, the prevalence of optimal 25(OH)D_3_ levels (>75 nmol/L) was relatively high among HIV-infected Ugandan patients with and without TB [46].

While many studies have been performed to investigate vitD_3_ status in PTB patients and controls, few studies have addressed differences in 25(OH)D_3_ levels in PTB to patients with extra-pulmonary forms of TB. In this study, we found that vitD_3_ levels were relatively lower in patients with PTB, and also pleural TB, compared with LNTB. Local TB lymphadenitis is a common and relatively mild form of TB compared with PTB. Instead, significantly lower vitD_3_ levels were previously shown in plasma and particularly in pleural fluid from patients with pleural TB compared with healthy controls [47]. It has been described that low vitD_3_ levels and severe VDD were more common in extra-pulmonary TB compared with PTB, suggesting that VDD was primarily a risk factor for extra-pulmonary disease [3]. However, data from local LNTB only, was not presented in this study [3]. We have previously shown that PTB patients with severe VDD have down-modulated LL-37 mRNA and protein expression in granulomatous TB lesions in the lung [48]. Similarly, results from this study suggested that LL-37 was elevated in the peripheral circulation in patients with PTB and pleural TB, likely as a consequence of an acute phase response, rather than the induction of antimicrobial LL-37 at the site of Mtb infection in the lung. Instead, local LL-37 expression in LNTB correlated with relatively higher 25(OH)D_3_ levels in plasma and LL-37 was confined to the granulomatous lesions in the lymph nodes. Interestingly, in vitro vitD_3_ stimulation of human macrophages from PTB patients showed that LL-37 expression was more significantly induced in patients with non-cavitary TB compared to patients with more severe cavitary TB [49]. Thus, it is tempting to speculate that advanced or poorly controlled intrathoracic TB is more often associated with a low vitD_3_ status.

Apart from vitD_3_ status, it is also likely that genetic variations in the intracellular vitamin D receptor (VDR) may affect the susceptibility to TB infection and that VDR polymorphisms are affected by ethnicity [50]. Some polymorphisms may result in longer, more inactive VDR proteins, while other genetic variants could affect VDR mRNA stability [50]. Meta-analyses suggest that the VDR *FokI* polymorphism is associated with an increased risk of pulmonary TB in East Asians [51], while the *t* allele of the VDR *TaqI* polymorphism is significantly associated with an increased TB risk in South and West Asians [52]. In addition, certain VDR polymorphisms also contribute to TB susceptibility when observed in combination with VDD [53]. Therefore, although we did not determine the VDR genotype in this study cohort, genetic variations in the VDR and other proteins involved in the vitD_3_ signaling pathway, such as the vitD_3_ binding protein, may affect the susceptibility to TB.

## 5. Conclusions

In conclusion, this study underlines that the extent and degree of VDD can vary significantly between different study populations based on several independent factors that may influence the susceptibility to TB. While it is likely that VDD constitutes a risk to develop active TB, VDD may also affect, or be affected by, the severity of TB disease. Thus, more severe forms of intrathoracic TB may be associated with lower vitD_3_ status and lower expression of antimicrobial LL-37 at the site of Mtb infection. Instead, higher systemic levels of LL-37 may be a consequence of reduced TB control and enhanced pathological inflammation.

## Figures and Tables

**Table 1:** Clinical demographics of the study participants.

Variable	Total Active TB (*n* = 77)	PTB (*n* = 32)	Pleural TB (*n* = 20)	LNTB (*n* = 25)	Ethiopian Non-TB Controls (*n* = 78)	Swedish Non-TB Controls (*n* = 62)
Gender:						
Male (M)–No (%)	42 (55%)	18 (56%)	10 (50%)	14 (56%)	41 (54%)	17 (27%)
Female (F)–No (%)	35 (45%)	14 (44%)	10 (50%)	11 (44%)	35 (46%)	45 (73%)
Age, median (range)	27 (18–72)	28.5 (18–54)	27 (19–72)	25.5 (18–57)	29 (18–68)	51.5 (21–72)
HIV infection, No (%)	24 (31%)	11 (34%)	5 (25%)	8 (32%)	32 (41%)	0 (0%)
Positive TST, No (%)	65 (84%)	26 (81%)	16 (80%)	23 (92%)	40 (51%)	ND
Weight loss, No (%)	55 (71%)	28 (88%)	15 (75%)	12 (48%)	ND	ND
Occupation, No (%)	33 (43%)	17 (53%)	9 (45%)	7 (28%)	ND	ND
Other illness, No (%) ^a^	6 (8%)	3 (9%)	2 (10%)	1 (4%)	ND	ND
25(OH)D_3_ nmol/L (median)	38.5	29.0	39.0	48.0	35.0	51.0
25(OH)D_3_ < 25 nmol/L–No (%)	15 (20%)	8 (25%)	4 (20%)	3 (12%)	14 (18%)	4 (6%)
25(OH)D_3_ < 50 nmol/L–No (%) ^b^	50 (66%)	24 (75%)	11 (58%)	15 (60%)	63 (81%)	31 (50%)
25(OH)D_3_ 50–75 nmol/L–No (%)	23 (30%)	7 (22%)	7 (37%)	9 (36%)	15 (19%)	26 (42%)
25(OH)D_3_ > 75 nmol/L–No (%)	3 (4%)	1 (3%)	1 (5%)	1 (4%)	0 (0%)	5 (8%)

PTB, pulmonary TB; LNTB, lymph node TB; TST, Tuberculin Skin Test; ND, No Data/Not Determined. ^a^ Other illnesses included diabetes, asthma, psychological problems, breast lesion, herpes zoster, and gastritis. ^b^ The proportion of Ethiopian subjects, including both active TB patients and non-TB controls, with VDD (<50 nmol/L) is 73%.

## References

[1] World Health Organization (WHO) (2016). Global Tuberculosis Report 2016.

[2] Zittermann A., Pilz S., Hoffmann H., Marz W. (2016). Vitamin D and airway infections: A European perspective. Eur. J. Med. Res..

[3] Pareek M., Innes J., Sridhar S., Grass L., Connell D., Woltmann G., Wiselka M., Martineau A.R., Kon O.M., Dedicoat M. (2015). Vitamin d deficiency and tb disease phenotype. Thorax.

[4] Gois P.H.F., Ferreira D., Olenski S., Seguro A.C. (2017). Vitamin d and infectious diseases: Simple bystander or contributing factor?. Nutrients.

[5] Schaaf H.S., Nel E.D., Beyers N., Gie R.P., Scott F., Donald P.R. (1996). A decade of experience with mycobacterium tuberculosis culture from children: A seasonal influence on incidence of childhood tuberculosis. Tuber. Lung Dis..

[6] Martineau A.R., Nhamoyebonde S., Oni T., Rangaka M.X., Marais S., Bangani N., Tsekela R., Bashe L., de Azevedo V., Caldwell J. (2011). Reciprocal seasonal variation in vitamin d status and tuberculosis notifications in cape town, south Africa. Proc. Natl. Acad. Sci. USA.

[7] Holick M.F., Binkley N.C., Bischoff-Ferrari H.A., Gordon C.M., Hanley D.A., Heaney R.P., Murad M.H., Weaver C.M., Endocrine S. (2011). Evaluation, treatment, and prevention of vitamin D deficiency: An endocrine society clinical practice guideline. J. Clin. Endocrinol. Metab..

[8] Martin C.A., Gowda U., Renzaho A.M. (2016). The prevalence of vitamin d deficiency among dark-skinned populations according to their stage of migration and region of birth: A meta-analysis. Nutrition.

[9] Stead W.W., Senner J.W., Reddick W.T., Lofgren J.P. (1990). Racial differences in susceptibility to infection by mycobacterium tuberculosis. N. Engl. J. Med..

[10] Lang P.O., Aspinall R. (2017). Vitamin d status and the host resistance to infections: What it is currently (not) understood. Clin. Ther..

[11] Martineau A.R., Wilkinson K.A., Newton S.M., Floto R.A., Norman A.W., Skolimowska K., Davidson R.N., Sorensen O.E., Kampmann B., Griffiths C.J. (2007). IFN-gamma- and TNF-independent vitamin D-inducible human suppression of mycobacteria: The role of cathelicidin LL-37. J. Immunol..

[12] Choi K.Y., Chow L.N., Mookherjee N. (2012). Cationic host defence peptides: Multifaceted role in immune modulation and inflammation. J. Innate Immun..

[13] Rivas-Santiago B., Hernandez-Pando R., Carranza C., Juarez E., Contreras J.L., Aguilar-Leon D., Torres M., Sada E. (2008). Expression of cathelicidin LL-37 during mycobacterium tuberculosis infection in human alveolar macrophages, monocytes, neutrophils, and epithelial cells. Infect. Immun..

[14] Liu P.T., Stenger S., Tang D.H., Modlin R.L. (2007). Cutting edge: Vitamin d-mediated human antimicrobial activity against mycobacterium tuberculosis is dependent on the induction of cathelicidin. J. Immunol..

[15] Rekha R.S., Rao Muvva S.S., Wan M., Raqib R., Bergman P., Brighenti S., Gudmundsson G.H., Agerberth B. (2015). Phenylbutyrate induces LL-37-dependent autophagy and intracellular killing of mycobacterium tuberculosis in human macrophages. Autophagy.

[16] Torres-Juarez F., Cardenas-Vargas A., Montoya-Rosales A., Gonzalez-Curiel I., Garcia-Hernandez M.H., Enciso-Moreno J.A., Hancock R.E., Rivas-Santiago B. (2015). LL-37 immunomodulatory activity during mycobacterium tuberculosis infection in macrophages. Infect. Immun..

[17] Elenius V., Palomares O., Waris M., Turunen R., Puhakka T., Ruckert B., Vuorinen T., Allander T., Vahlberg T., Akdis M. (2017). The relationship of serum vitamins a, d, e and ll-37 levels with allergic status, tonsillar virus detection and immune response. PLoS ONE.

[18] Bivona G., Agnello L., Ciaccio M. (2017). Vitamin d and immunomodulation: Is it time to change the reference values?. Ann. Clin. Lab. Sci..

[19] Hoe E., Nathanielsz J., Toh Z.Q., Spry L., Marimla R., Balloch A., Mulholland K., Licciardi P.V. (2016). Anti-inflammatory effects of vitamin d on human immune cells in the context of bacterial infection. Nutrients.

[20] Ashenafi S., Aderaye G., Bekele A., Zewdie M., Aseffa G., Hoang A.T., Carow B., Habtamu M., Wijkander M., Rottenberg M. (2014). Progression of clinical tuberculosis is associated with a Th2 immune response signature in combination with elevated levels of SOC S3. Clin. Immunol..

[21] Ashenafi S., Aderaye G., Zewdie M., Raqib R., Bekele A., Magalhaes I., Lema B., Habtamu M., Rekha R.S., Aseffa G. (2013). BCG-specific igg-secreting peripheral plasmablasts as a potential biomarker of active tuberculosis in HIV negative and HIV positive patients. Thorax.

[22] Bergman P., Norlin A.C., Hansen S., Rekha R.S., Agerberth B., Bjorkhem-Bergman L., Ekstrom L., Lindh J.D., Andersson J. (2012). Vitamin D_3_ supplementation in patients with frequent respiratory tract infections: A randomised and double-blind intervention study. BMJ Open.

[23] Livak K.J., Schmittgen T.D. (2001). Analysis of relative gene expression data using real-time quantitative PCR and the 2(-delta delta c(t)) method. Methods.

[24] Andersson J., Samarina A., Fink J., Rahman S., Grundstrom S. (2007). Impaired expression of perforin and granulysin in CD8+ T cells at the site of infection in human chronic pulmonary tuberculosis. Infect. Immun..

[25] Brighenti S., Andersson J. (2012). Local immune responses in human tuberculosis: Learning from the site of infection. J. Infect. Dis..

[26] Campbell M.S., Lovell M.A., Gorbsky G.J. (1995). Stability of nuclear segments in human neutrophils and evidence against a role for microfilaments or microtubules in their genesis during differentiation of HL60 myelocytes. J. Leukoc. Biol..

[27] Nnoaham K.E., Clarke A. (2008). Low serum vitamin d levels and tuberculosis: A systematic review and meta-analysis. Int. J. Epidemiol..

[28] Zeng J., Wu G., Yang W., Gu X., Liang W., Yao Y., Song Y. (2015). A serum vitamin d level <25 nmol/L pose high tuberculosis risk: A meta-analysis. PLoS ONE.

[29] Keflie T.S., Nolle N., Lambert C., Nohr D., Biesalski H.K. (2015). Vitamin d deficiencies among tuberculosis patients in Africa: A systematic review. Nutrition.

[30] Huang S.J., Wang X.H., Liu Z.D., Cao W.L., Han Y., Ma A.G., Xu S.F. (2017). Vitamin d deficiency and the risk of tuberculosis: A meta-analysis. Drug Des. Devel. Ther..

[31] Workineh M., Mathewos B., Moges B., Gize A., Getie S., Stendahl O., Schon T., Abate E. (2017). Vitamin D deficiency among newly diagnosed tuberculosis patients and their household contacts: A comparative cross-sectional study. Arch. Public Health.

[32] Tessema B., Moges F., Habte D., Hiruy N., Yismaw S., Melkieneh K., Kassie Y., Girma B., Melese M., Suarez P.G. (2017). Vitamin D deficiency among smear positive pulmonary tuberculosis patients and their tuberculosis negative household contacts in northwest Ethiopia: A case-control study. Ann. Clin. Microbiol. Antimicrob..

[33] Arnedo-Pena A., Juan-Cerdan J.V., Romeu-Garcia M.A., Garcia-Ferrer D., Holguin-Gomez R., Iborra-Millet J., Pardo-Serrano F. (2015). Vitamin D status and incidence of tuberculosis infection conversion in contacts of pulmonary tuberculosis patients: A prospective cohort study. Epidemiol. Infect..

[34] Khandelwal D., Gupta N., Mukherjee A., Lodha R., Singh V., Grewal H.M., Bhatnagar S., Singh S., Kabra S.K., Delhi Pediatric T.B.S.G. (2014). Vitamin d levels in Indian children with intrathoracic tuberculosis. Indian J. Med. Res..

[35] Musarurwa C., Zijenah L.S., Duri D.Z., Mateveke-Dangaiso K., Mhandire K., Chipiti M.M., Munjoma M.W., Mujaji W.B. (2017). Association of high serum vitamin D concentrations with active pulmonary TB in an HIV co-endemic setting, Harare, Zimbabwe. BMC Infect. Dis..

[36] Owolabi O., Agbla S., Owiafe P., Donkor S., Togun T., Sillah A.K., Ota M.O., Sutherland J.S. (2016). Elevated serum 25-hydroxy (OH) vitamin D levels are associated with risk of TB progression in Gambian adults. Tuberculosis.

[37] Friis H., Range N., Changalucha J., Praygod G., Jeremiah K., Faurholt-Jepsen D., Krarup H., Molgaard C., Andersen A. (2013). Vitamin D status among pulmonary TB patients and non-TB controls: A cross-sectional study from Mwanza, Tanzania. PLoS ONE.

[38] Wejse C., Olesen R., Rabna P., Kaestel P., Gustafson P., Aaby P., Andersen P.L., Glerup H., Sodemann M. (2007). Serum 25-hydroxyvitamin d in a west African population of tuberculosis patients and unmatched healthy controls. Am. J. Clin. Nutr..

[39] Webb A.R., Kline L., Holick M.F. (1988). Influence of season and latitude on the cutaneous synthesis of vitamin D3: Exposure to winter sunlight in Boston and Edmonton will not promote vitamin D3 synthesis in human skin. J. Clin. Endocrinol. Metab..

[40] Fares A. (2011). Seasonality of tuberculosis. J. Glob. Infect. Dis..

[41] Friis H., Range N., Pedersen M.L., Molgaard C., Changalucha J., Krarup H., Magnussen P., Soborg C., Andersen A.B. (2008). Hypovitaminosis d is common among pulmonary tuberculosis patients in Tanzania but is not explained by the acute phase response. J. Nutr..

[42] Korthals Altes H., Kremer K., Erkens C., van Soolingen D., Wallinga J. (2012). Tuberculosis seasonality in the Netherlands differs between natives and non-natives: A role for vitamin d deficiency?. Int. J. Tuberc. Lung Dis..

[43] Feleke Y., Abdulkadir J., Mshana R., Mekbib T.A., Brunvand L., Berg J.P., Falch J.A. (1999). Low levels of serum Calcidiol in an African population compared to a north European population. Eur J Endocrinol.

[44] Nansera D., Graziano F.M., Friedman D.J., Bobbs M.K., Jones A.N., Hansen K.E. (2011). Vitamin d and calcium levels in Ugandan adults with human immunodeficiency virus and tuberculosis. Int. J. Tuberc. Lung Dis..

[45] Steenhoff A.P., Redwood A., Pettifor J.M., Hove J., Bisson G.P., Mosepele M., Pusoesele P., Thakur R., Kovarik C., Gross R. (2012). Vitamin D status in HIV-infected patients with and without tuberculosis: A pilot study. J. Acquir. Immune Defic. Syndr..

[46] Conesa-Botella A., Goovaerts O., Massinga-Loembe M., Worodria W., Mazakpwe D., Luzinda K., Mayanja-Kizza H., Colebunders R., Kestens L., TB IRIS Study Group (2012). Low prevalence of vitamin d deficiency in Ugandan HIV-infected patients with and without tuberculosis. Int. J. Tuberc. Lung Dis..

[47] Srinivasan A., Syal K., Banerjee D., Hota D., Gupta D., Kaul D., Chakrabarti A. (2013). Low plasma levels of cholecalciferol and 13-cis-retinoic acid in tuberculosis: Implications in host-based chemotherapy. Nutrition.

[48] Rahman S., Rehn A., Rahman J., Andersson J., Svensson M., Brighenti S. (2015). Pulmonary tuberculosis patients with a vitamin D deficiency demonstrate low local expression of the antimicrobial peptide LL-37 but enhanced FoxP3+ regulatory T cells and IgG-secreting cells. Clin Immunol.

[49] Afsal K., Harishankar M., Banurekha V.V., Meenakshi N., Parthasarathy R.T., Selvaraj P. (2014). Effect of 1,25-dihydroxy vitamin D3 on cathelicidin expression in patients with and without cavitary tuberculosis. Tuberculosis.

[50] Uitterlinden A.G., Fang Y., Van Meurs J.B., Pols H.A., Van Leeuwen J.P. (2004). Genetics and biology of vitamin d receptor polymorphisms. Gene.

[51] Lee Y.H., Song G.G. (2015). Vitamin D receptor gene Foki, Taqi, Bsmi, and Apai polymorphisms and susceptibility to pulmonary tuberculosis: A meta-analysis. Genet. Mol. Res..

[52] Cao Y., Wang X., Cao Z., Cheng X. (2015). Association of vitamin d receptor gene Taqi polymorphisms with tuberculosis susceptibility: A meta-analysis. Int. J. Clin. Exp. Med..

[53] Wilkinson R.J., Llewelyn M., Toossi Z., Patel P., Pasvol G., Lalvani A., Wright D., Latif M., Davidson R.N. (2000). Influence of vitamin d deficiency and vitamin D receptor polymorphisms on tuberculosis among Gujarati Asians in west London: A case-control study. Lancet.

